# The first see-through frog created by breeding: description, inheritance patterns, and dermal chromatophore structure

**DOI:** 10.1038/srep24431

**Published:** 2016-04-15

**Authors:** Masayuki Sumida, Mohammed Mafizul Islam, Takeshi Igawa, Atsushi Kurabayashi, Yukari Furukawa, Naomi Sano, Tamotsu Fujii, Norio Yoshizaki

**Affiliations:** 1 Institute for Amphibian Biology, Graduate School of Science, Hiroshima University, Higashihiroshima 739-8526, Japan; 2 Graduate School for International Development and Cooperation, Hiroshima University, Higashihiroshima 739-8529, Japan; 3 Faculty of Human Culture & Science, Prefectural University of Hiroshima, Hiroshima 734-8558, Japan; 4 Faculty of Applied Biological Science, Gifu University, Yanagido, Gifu 501-1193, Japan

## Abstract

We have succeeded in creating see-through frogs from natural color mutants of the Japanese brown frog *Rana japonica*, which usually possesses an ochre or brown back; this coloration enables the organs, blood vessels, and eggs to be observed through the skin without performing dissection. We crossed two kinds of recessive color mutant (black-eyed and gray-eyed) frogs through artificial insemination, and F2 offspring produced frogs whose skin is translucent throughout the life cycle. Three kinds of dermal chromatophores—xanthophores, iridophores, and melanophores—are observed in a layered arrangement in the skin of wild-type frogs, but few chromatophores were present in the skin of the see-through frogs. The translucent skin enables observation of organ growth and cancer formation and progression in the animal, which can be monitored over its entire life without the need for dissection. See-through frogs thus provide a useful animal model for environmental, medical, and biological research.

Dissection, particularly when performed in schools, has become increasingly controversial in much of the world. However, if the internal organs of an animal were clearly visible through the skin over the animal’s entire life history, then there would be a greatly reduced need for dissection. In frogs, the skin is generally covered with dermal chromatophore units consisting of xanthophores, iridophores, and melanophores^1^, which block the view of the internal organs through the skin. Although some small transparent fish species appropriate for experimental use have been previously reported^2^,^3^, see-through tetrapods have not yet been developed. Several species of see-through glassfrogs belonging to the family Centrolenidae have recently been found in tropical wet forests and premontane rainforests in Central and South America; these frogs possess transparent parietal and cardial peritonea^4^,^5^. However, the creation of a see-through frog that is translucent over the entire body—to allow observation of organs, blood vessels, and eggs through the skin—remains a goal, with the primary purpose of providing an experimental model animal where organs can be observed over the entire life cycle without dissection.

The black-eyed and gray-eyed recessive color mutants seen in several frog species lack the normal iridophores and melanophores, respectively, resulting in pale or albino frogs^6^,^7^,^8^,^9^,^10^,^11^,^12^,^13^. To create translucent frogs from these two color mutants of the Japanese brown frog *Rana japonica*^12^,^14^,^15^,^16^, and to clarify the mode of inheritance in the resulting see-through frog, we crossed two mutant frogs through artificial insemination. F2 offspring produced frogs with skin that remains translucent throughout life. We also observed the dermal chromatophores in the skin of wild-type, black-eyed, gray-eyed, and see-through frogs with an electron microscope to examine the microstructure of the dermal chromatophores in the dorsal skin of see-through frogs. The present paper reports our experiments in detail, although a brief note on this topic has been previously published as a News-in-Brief (*Nature*)^17^.

## Results

### Creation of see-through frogs

**Figure 1:**
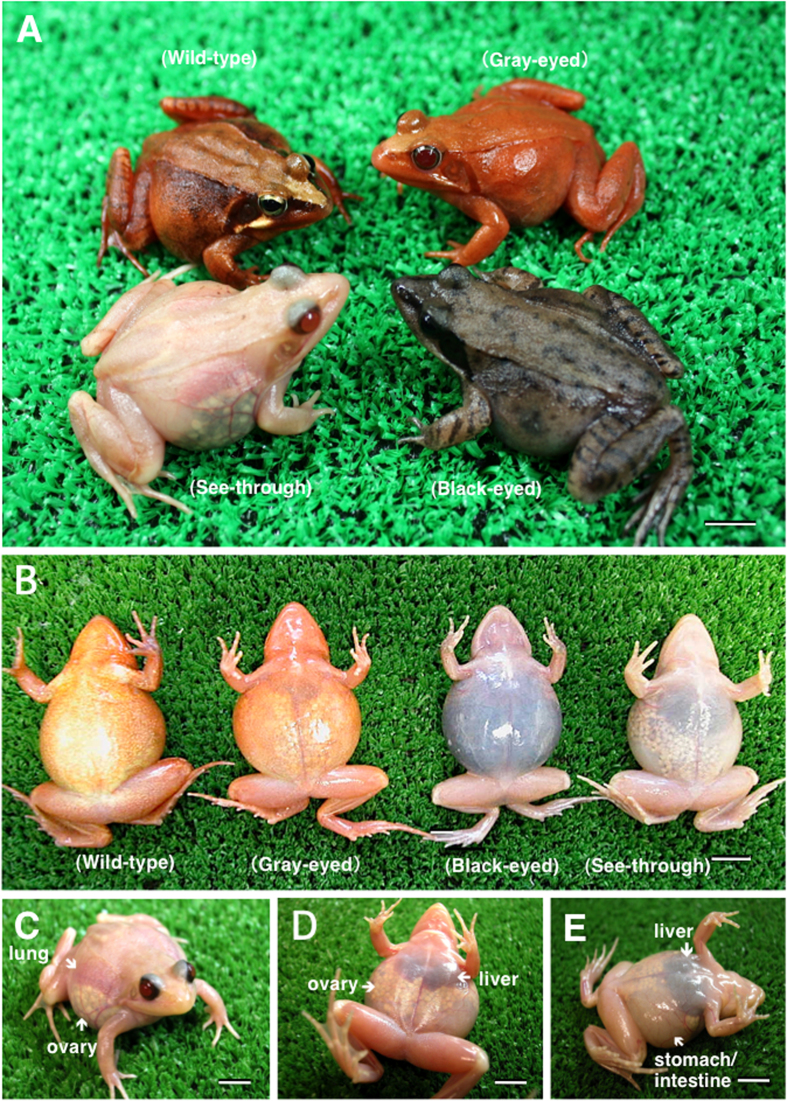
See-through, two color mutant, and wild-type adult *Rana japonica* frogs. (**A**) Dorsolateral view of four types. (**B**) Ventral view of four types. (**C**) See-through frog (frontal view). (**D**) See-through frog (ventral view). (**E**) See-through frog (ventrolateral view). (Scale bar 1 cm)

**Figure 2:**
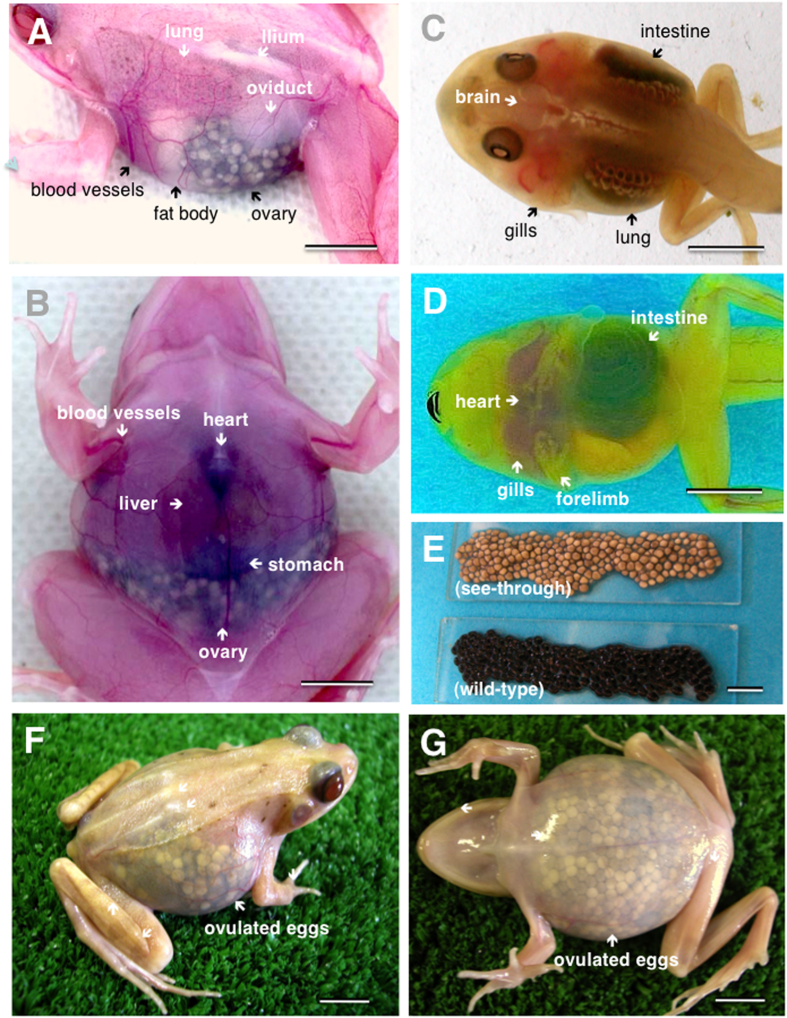
Internal organs of live see-through frogs and tadpoles visible through the translucent skin. (**A**) Breathing see-through frog (dorsolateral view). (**B**) Heart beating see-through frog (ventral view). (**C**) See-through tadpole (dorsal view). (**D**) See-through tadpole (ventral view). (**E**) Eggs of see-through and wild-type frogs (just after insemination). (**F**) Ovulated see-through frog (lateral view). (**G**) Ovulated see-through frog (ventral view). (Scale bar 1 cm)

Upon crossing two color mutant (gray-eyed and black-eyed) frogs with recessive genes through artificial insemination, all of the offspring appeared normal (wild-type) due to the presence of dominant genes (Fig. 1A,B, Table 1). However, brother-sister mating among the wild-type offspring led to frogs with translucent skin from the tadpole stage, thereby successfully generating see-through frogs with visible viscera. The see-through frog is the first transparent four-legged animal to be developed artificially. Organs such as the lungs, liver, heart, ovaries, stomach, intestines, oviduct, and fat bodies are visible through the translucent skin when the animal is viewed ventrally or laterally (Fig. 1C–E, Fig. 2A,B). Dramatic changes during metamorphosis such as gill disappearance, lung development, intestinal shortening, and emergence of forelimbs can be easily viewed in the developmental process of metamorphosis (Fig. 2C,D). See-through frogs also allow observation of ovulation (Fig. 2F,G). Ovulation was induced in mature female see-through frogs by injecting a suspension of bullfrog pituitary extract into the abdominal cavity. Deposited see-through frog eggs were white in color, as opposed to the normal black color of wild-type eggs (Fig. 2E). All of these processes are visible through the skin, without dissecting the tadpole or the frog. Videos of the see-through frogs and tadpoles produced in this study are available online (adult frog, https://www.youtube.com/watch?v=3TumwZ40LQM; tadpole https://www.youtube.com/watch?v=tYRjZtaxOgg).

As shown in Table 1, the production rates of see-through frogs depended on the combination of parental genotypes following the law of segregation (9:3:3:1, χ^2^ = 2.7445, *P* = 0.433; 3:3:1:1, χ^2^ = 4.8660, *P* = 0.182). The most effective crossing combinations were between Bbgg female and BbGg male parents. See-through frogs produced by the F2 generation had low viability, probably due to the presence of two recessive genes.

### Microstructure of dermal chromatophores in the dorsal skin

**Figure 3:**
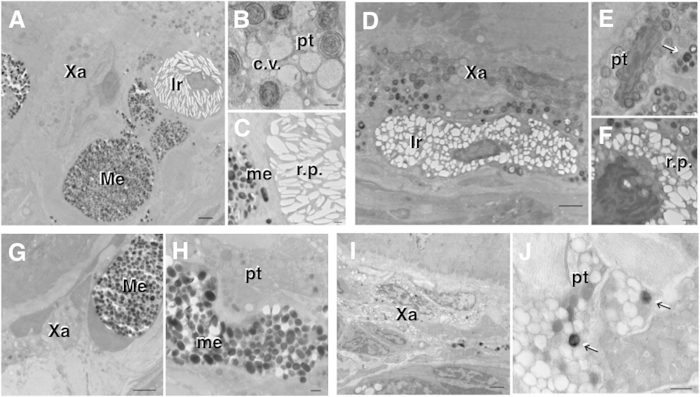
Electron microphotographs of dermal chromatophores in the dorsal skin. (**A–C**) Wild-type frog. (**D–F**) Gray-eyed frog. (**G,H**) Black-eyed frog. (**I,J**) See-through frog. Me: melanophore, Xa: xanthophore, Ir: iridophore, me: melanosome, pt: pterinosome, c.v.: carotenoide vesicle, r.p.: reflecting platelet. Arrows indicate melanosome-like structures. (Scale bar **A,D,G,I**: 2 μm; **B,C,E,F,H,J**: 500 nm)

Three kinds of dermal chromatophores—xanthophores, iridophores, and melanophores—were observed in a layered arrangement in the skin of wild-type frogs (Fig. 3A). Pterinosomes and carotenoid vesicles were found in the xanthophores (Fig. 3B), and reflecting platelets and melanosomes were observed in the iridophores and melanophores, respectively (Fig. 3C). The skin of gray-eyed frogs contained no melanophores, and had only xanthophores and iridophores (Fig. 3D). Normal pterinosomes and many abnormal round reflecting platelets were observed in the xanthophores and iridophores, respectively (Fig. 3E,F). The skin of black-eyed frogs contained xanthophores and melanophores, but not iridophores (Fig. 3G). Normal melanosomes were found in the melanophores, whereas immature pterinosomes or carotenoid vesicles were observed in the xanthophores (Fig. 3H). In the skin of see-through frogs, there was a low number of chromatophores (Fig. 3I), but immature xanthophore-like cells containing melanosome-like structures were observed (Fig. 3J).

## Discussion

The see-through frogs produced in this study allowed us to observe changes in the internal tissues and organs in detail, externally, in both early development and senescence, to evaluate the effects of chemicals on the viscera and bones in a simple and inexpensive manner, to view the development and progression of cancer, and to assess the effects of toxins over time. See-through frogs do not need to be dissected to perform such viewing and therefore enable the repeated ongoing observation of the viscera over the entire life course in a single frog. Furthermore, the dramatic changes in organs that occur during the metamorphosis of tadpoles into adult frogs can be easily seen.

Genetic engineering of see-through frogs is an approach that could be used to produce see-through, fluorescent frogs. In the near future, we plan to produce frogs that are both see-through and fluorescent by injecting fluorescent GFP-tagged genes into see-through frogs, causing the frogs to fluoresce to indicate expression of the tagged gene^18^. This could be used for many purposes, for example, showing when cancer starts. See-through frogs can also be used as experimental model animals in various fields, such as medicine, veterinary medicine, biology, and education. Commercial production and marketing applications for see-through frogs as ornamental pets is another possibility, and the frogs have been nicknamed “*sukeru-pyon*”, combining the Japanese words for “see-through” and “frog”.

The see-through frogs produced in the present study appear pale when viewed dorsally, as xanthophores remain in the dorsal skin. However, these frogs are almost transparent when viewed ventrally, because there are little to no xanthophores in the ventral skin. If we could obtain color mutants that lack xanthophores, we would be able to produce completely see-through frogs by using these mutants to produce triple mutants lacking genes for all three chromatophores. In fact, variant blue ranid frogs have been found in several frog species^19^,^20^,^21^,^22^, but we have not yet found this kind of color mutant in the Japanese brown frog *Rana japonica*. We hope to create completely see-through frogs in the near future. It is worth noting that it is unrealistic to apply the present method to mammals such as mice, given the differences in skin structure, although the development of whole-body imaging at single-cell resolution has recently enabled system-level approaches to studying cellular circuits in mice by using tissue decolorization^23^. See-through frogs are not stable enough to produce offspring, probably due to inbreeding depression caused by two recessive genes. This is another problem that must be addressed in the future by using outbreeding, via artificial insemination, to avoid inbreeding depression.

Immature xanthophore-like cells containing melanosome-like structures were observed in the skin of the see-through frogs. Some evidence suggesting the conversion of xanthophores into melanophores *in vitro* and *in vivo* has been reported^24^,^25^,^26^,^27^, although it is difficult to follow the conversion of a single xanthophore precisely, and there has been no conclusive evidence demonstrating the conversion of a xanthophore into a melanophore *in vivo*. Evidence that melanosomes and pterinosomes are homologous organelles has been reported in several studies^28^,^29^,^30^,^31^, and similarities exist in the morphological characteristics between the two. Ide^32^ demonstrated the conversion of amphibian xanthophores into melanophores *in vitro*. In the cloned xanthophore, pre-existing pterinosomes disappeared and melanosomes were formed instead during proliferation. Ide considered these melanosomes to originate de novo from melanosomes via premelanosomes, because of the presence of premelanosomes in the melanized xanthophores. The results of the present study also suggest that xanthophores may be converted into melanophores, demonstrating the de novo formation of melanosomes via premelanosomes.

Another possible explanation may be that xanthophores contain melanosome-like organelles, but not transdifferentiated chromatophores, as melanosome-like structures were also observed in xanthophores in the skin of gray-eyed frogs (Fig. 3D–F). According to Bagnara^33^, pigment cells occasionally occur that contain not only their definitive organelle type, but also the pigment organelles of other pigment cell types. Chromatophore mosaicisms, where different types of pigment granules are contained within a single pigment cell, have been reported in several kinds of frogs^13^,^33^,^34^,^35^,^36^. Some mosaic or polychromatic hybrid chromatophores appear to possess organelles characteristic of other chromatophores, for example, melanophores containing pterinosomes or xanthophores containing melanosomes^34^. These mosaic cells are given compound names, such as “irido-melanophores” for chromatophores that reflect light and produce melanin^35^,^36^. The pigment granules contained in a single pigment cell are variable, and mosaic chromatophores that contain three types of pigment granules have also been found^13^,^35^. Melanophores, xanthophores, and iridophores are fundamentally distinct in their appearance, composition, and function, but all migrate from their site of origin at the neural crest to populate the integument. Their respective pigments—melanins, pteridines, and purines—are found in organelles designated as melanosomes, pterinosomes, and reflecting platelets, respectively. These organelles are all derived from endoplasmic reticular vesicles. This commonality is in keeping with the hypothesis of the common origin of pigment cells from a stem cell containing a primordial organelle with the potential to become any derived pigmentary organelle^37^. 

In light of these findings, further studies will be necessary to clarify the processes involved in chromatophore formation in more detail, and to examine the fine structure of pigment organelles by observing pigment cell development in see-through tadpoles and frogs using electron microscopy.

This study has received a patent under “Creation and Use of See-through Frogs”, application number (2006-203987), open number (2008-029223) on application date (2006.7.26), open date (2008.2.14), applicant (Hiroshima University), creator (Masayuki Sumida).

## Methods

### Animal ethics

All animal experiments were performed in accordance with the approved guidelines of Hiroshima University regarding protection and management of animals and standards for the breeding, safekeeping, and minimization of suffering of experimental animals. All experimental protocols were approved by the Hiroshima University Animal Experiment Committee, and were in keeping with the basic guidelines of Hiroshima University regarding animal experiments (Approval numbers: 24-116, 26-209).

### Creation of see-through frogs

We used two color mutants (black-eyed and gray-eyed) that were originally caught in the field, and then bred and maintained in the Institute for Amphibian Biology, Hiroshima University^12^,^22^. These mutants were crossed by artificial insemination to produce frogs homozygous at both gene loci over several generations. Ovulation was accelerated by injecting a suspension of bullfrog pituitary into the abdominal cavity. Fertilization was always performed artificially. Tadpoles were fed boiled spinach and metamorphosed frogs were fed crickets^38^,^39^.

### Microstructure of dermal chromatophores in the skin

We observed the dermal chromatophores in the dorsal skin of wild-type, black-eyed, gray-eyed, and see-through adult frogs with an electron microscope to examine the microstructure of the skin of see-through frogs. Pieces of the dorsal skins removed from each of these frogs were cut into minute pieces in cold 0.1 M phosphate buffer (pH 7.4) containing 3% glutaraldehyde and kept in the same solution for 2 h after renewal of the fluid. The minute pieces were then postfixed in 0.1 M phosphate buffer (pH 7.4) containing 2% osmic acid for 2 h. These fixation procedures were performed at 2–4 °C. The fixed samples were dehydrated in an ethanol series and embedded in Epon 812. Sections were cut on a Porter-Blum MT-1 ultramicrotome with a glass knife and double stained with saturated uranyl acetate and alkaline lead citrate. Observations were made using a Hitachi Hs-8 electron microscope.

## Additional Information

**How to cite this article**: Sumida, M. *et al.* The first see-through frog created by breeding: description, inheritance patterns, and dermal chromatophore structure. *Sci. Rep.* **6**, 24431; doi: 10.1038/srep24431 (2016).

## Figures and Tables

**Table 1:**
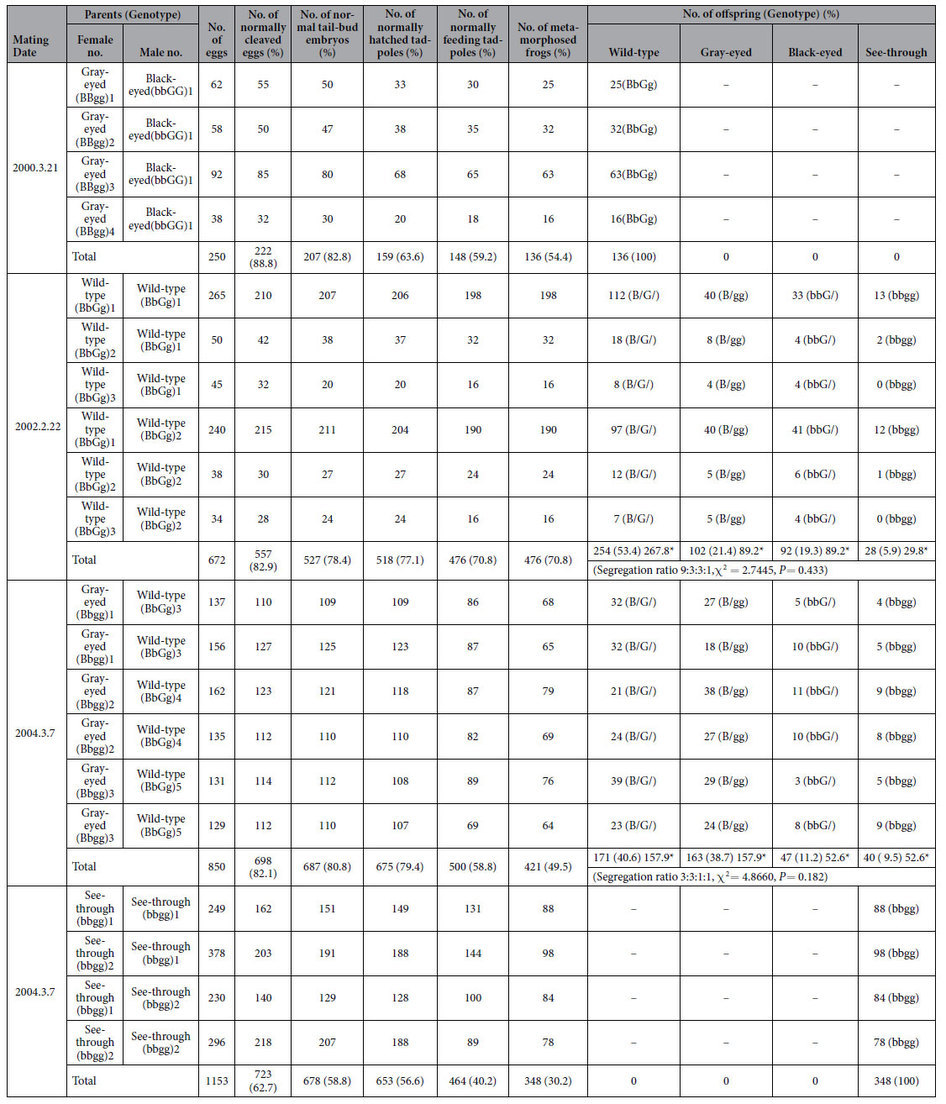
Crossing experiments for creating see-through frogs.

Asterisks show the expected values from the segregation ratios.

## References

[b1] Bagnara J. T., Taylor J. D. & Hadley M. E. The dermal chromatophore unit. J. Cell Biol. 38, 67–79 (1968). 5691979 10.1083/jcb.38.1.67 PMC2107474

[b2] Wakamatsu Y., Pristyazhnyuk S., Kinoshita M., Tanaka M. & Ozato K. The see-through medaka: A fish model that is see-through throughout life. Proc. Natl. Acad. Sci. USA 98, 10046–10050 (2001). 11526229 10.1073/pnas.181204298 PMC56912

[b3] Akiyama S.-I. & Tamaru Y. Development of transgenic technology using transparent goldfish. Abst. Ann. Meet. Soc. Biosci. Bioengineer. Japan 2010, 19 (2010).

[b4] Twomey E., Delia J. & Castroviejo-Fisher S. A review of Northern Peruvian glassfrogs (Centrolenidae), with the description of four new remarkable species. Zootaxa 3851, 1–87 (2014). 25112428 10.11646/zootaxa.3851.1.1

[b5] Kubicki B., Salazar S. & Puschendorf R. A new species of glassfrog, genus *Hyalinobatrachium* (Anura: Centrolenidae), from the Caribbean foothills of Costa Rica. Zootaxa 3920, 69–84 (2015). 25781240 10.11646/zootaxa.3920.1.4

[b6] Sally K. F., Ellinger M. S. & Murphy J. F. The pale mutation in *Bombina orientaris*: Effects on melanophores and xanthophores. J. Exp. Zool. 221, 125–129 (1982).

[b7] Bagnara J. T. Comparative anatomy and phylogeny of pigment cells in no mammalian tissues in *The pigmentary system*: Physiology and pathophysiology (eds Nordlung J. J. *et al.*) 9–40 (Oxford Univ. Press, 1998).

[b8] Nishioka M. & Ueda H. Genetics and morphology of 13 albino stocks in the *Rana nigromaculata* group. Sci. Rep. Lab. Amphibian Biol. Hiroshima Univ. 7, 1–121 (1985).

[b9] Nishioka M. & Ueda H. Electron-microscopic observation on the dermal chromatophores of normal frogs and three kinds of color variants in *Rhacophorus schlegelii*. Sci. Rep. Lab. Amphibian Biol. Hiroshima Univ. 7, 123–155 (1985).

[b10] Nishioka M. & Ueda H. Two kinds of black-eyed variants in *Hyla arborea japonica*. Sci. Rep. Lab. Amphibian Biol. Hiroshima Univ. 7, 157–179 (1985).

[b11] Nishioka M. & Ueda H. Non-inheritable color variants in *Rana brevipoda porosa*. Sci. Rep. Lab. Amphibian Biol. Hiroshima Univ. 7, 199–217 (1985).

[b12] Sumida M. & Nishioka M. Genetic linkage groups in the Japanese brown frog (*Rana japonica*). J. Hered. 91, 1–7 (2000). 10739117 10.1093/jhered/91.1.1

[b13] Ichikawa Y., Ohtani H. & Miura I. Pigment cells of amphibians. Electronmicroscope 38, 207–212 (2003). (in Japanese)

[b14] Sumida M. & Nishioka M. Geographic variability of sex-linked loci in the Japanese brown frog *Rana japonica*. Sci. Rep. Lab. Amphibian Biol. Hiroshima Univ. 13, 173–195 (1994).

[b15] Sumida M. Inheritance and linkage analysis of ten enzyme and blood protein loci in the Japanese brown frog *Rana japonica*. Biochem. Genet. 34, 375–388 (1996). 8978910 10.1007/BF00554413

[b16] Sumida M. & Nishioka M. Sex-linked genes and linkage maps in amphibians. Comp. Biochem. Physiol. 126, 257–270 (2000). 10.1016/s0305-0491(00)00204-2 10874173

[b17] Nature (News-in-Brief). See-through frogs created by breeding. Nature 449, 521 (2007).

[b18] Kinoshita M., Kani S., Ozato K. & Wakamatsu Y. Activity of the medaka translation elongation factor 1α-A promoter examined using the GFP gene as a reporter. Dev. Growth Differ. 42, 469–478 (2000). 11041488 10.1046/j.1440-169x.2000.00530.x

[b19] Berns M. W. & Narayan K. S. A histological and ultrastructural analysis of the dermal chromatophores of the variant ranid blue frog. J. Morph. 132, 169–179 (1970).

[b20] Nishioka M. Color variants induced by radiation and their inheritance in *Rana nigromaculata*. Sci. Rep. Lab. Amphibian Biol. Hiroshima Univ. 2, 25–89 (1977).

[b21] Nishioka M. & Ueda H. Blue variants in *Hyla arborea japonica*. Sci. Rep. Lab. Amphibian Biol. Hiroshima Univ. 7, 181–197 (1985).

[b22] Nishioka M. & Ueda H. Study on the color mutants in anurans. *Research report by 1988 fiscal Grant-in-Aid for Scientific Research (General Research A)* 1–26 (1989). (in Japanese)

[b23] Tainaka K. *et al.* Whole-body imaging with single-cell resolution by tissue decolorization. Cell 159, 911–924 (2014). 25417165 10.1016/j.cell.2014.10.034

[b24] Niu M. C. Further studies on the origin of amphibian pigment cells. J. Exp. Zool. 125, 199–220 (1954).

[b25] Loud A. V. & Mishima Y. The induction of melanization in goldfish scales with ACTH *in vivo*. J. Cell Biol. 18, 181–194 (1963). 19866632 10.1083/jcb.18.1.181 PMC2106279

[b26] Bagnara J. T. & Ferris W. Interrelationships of vertebrate chromatophores in Biology of normal and abnormal melanocytes (eds Kawamura T. *et al.*) 57–76 (Univ. Tokyo Press, 1971).

[b27] Chen S.-t., Wahn H., Turner W. A., Taylor J. D. & Tchen T. T. MSH, cyclic AMP, and melanocyte differentiation. Recent Progress in Hormone Research 30, 319–345 (1974). 4366787 10.1016/b978-0-12-571130-2.50012-2

[b28] Obika M. & Matsumoto J. Morphological and biochemical studies on amphibian bright-colored pigment cells and their pterinosomes. Exp. Cell Res. 52, 646–659 (1968). 4971656 10.1016/0014-4827(68)90504-1

[b29] Ide H. & Hama T. Tyrosinase activity of the melanin and pteridine granules in goldfish chromatophores. Biochim. Biophys. Acta 192, 200–204 (1969). 4984116 10.1016/0304-4165(69)90356-0

[b30] Yasutomi M. & Hama T. Ultramicroscopic study of the developmental change of the xanthophore in the frog, *Rana japonica* with special reference to pterinosomes. Dev. Growth Differ. 13, 141–149 (1971). 5147852 10.1111/j.1440-169x.1971.00141.x

[b31] Yasutomi M. & Hama T. Electron microscopic demonstration of tyrosinase in pterinosomes of the frog xanthophore, and the origin of pterinosomes. Dev. Growth Differ. 18, 289–299 (1976). 10.1111/j.1440-169X.1976.00289.x 37281460

[b32] Ide H. Transformation of amphibian xanthophores into melanophores in clonal culture. J. Exp. Zool. 203, 287–293 (1978).

[b33] Bagnara J. T. The neural crest as a source of stem cells in Developmental and evolutionary aspects of the neural crest (ed Maderson P.) 57–87 (New York Wiley, 1987).

[b34] Bagnara J. T. & Hadley M. E. Chromatophores and Color Change: The Comparative Physiology of Animal Pigmentation, 202 pp. (Prentice-Hall, Englewood Cliffs, N.J., 1973).

[b35] Bagnara J. T. *et al.* Common origin of pigment cells. Science 203, 410–415 (1979). 760198 10.1126/science.760198

[b36] Bagnara J. T. The emergence of pigment cell biology: a personal view. Pigment Cell Res. 12, 48–65 (1999). 10193681 10.1111/j.1600-0749.1999.tb00506.x

[b37] Bagnara J. T. Developmental aspects of vertebrate chromatophores. Integ. Comp. Biol. 23, 465–478 (1983).

[b38] Sumida M. Incipient intraspecific isolating mechanisms in the Japanese brown frog *Rana japonica*. J. Herpetol. 30, 333–346 (1996).

[b39] Sumida M., Ueda H. & Nishioka M. Reproductive isolating mechanisms and molecular phylogenetic relationships among Palearctic and Oriental brown frogs. Zool. Sci. 20, 567–580 (2003). 12777828 10.2108/zsj.20.567

